# Second derivative synchronous fluorescence determination of avanafil in the presence of its acid-induced degradation product aided by powerful Lean Six Sigma tools augmented with D-optimal design†

**DOI:** 10.1039/d0ra08216c

**Published:** 2021-01-19

**Authors:** Khalid A. M. Attia, Ahmad A. Mohamad, Mohamed S. Emara, Ahmed M. Abdel-Raoof, Mohamed A. Hasan, Ahmed W. Madkour, Ebrahim A. El-Desouky

**Affiliations:** Pharmaceutical Analytical Chemistry Dept., Faculty of Pharmacy, Al-Azhar University Nasr City Cairo 11751 Egypt Mohamed.emara83@yahoo.com +201002429533; Pharmaceutical Chemistry Department, Heliopolis University for Sustainable Development 3 Belbis Disert Road, Alsalam City Cairo 11777 Egypt

## Abstract

In this work, the quantitative determination of an erectile dysfunctional drug avanafil in the presence of its acid-induced degradation product was achieved *via* the application of a pre-optimized novel spectrofluorimetric method. The fluorescence emission wavelength was recorded at 370 and 407 nm, after being excited at 268 and 271 nm for avanafil and its acid-induced degradation product, respectively. Direct determination of avanafil based on its native fluorescence is restricted because the emission spectra of both components are heavily overlapped. Therefore, to overcome this constraint, a novel second derivative synchronous fluorescence method was evolved to eliminate this overlapping. The ideal determination wavelength was found to be 377 nm. Augmentation of lean six sigma (LSS) with response surface methodology (RSM) play a significant role in the development of robust specifications to ensure quality at the six sigma level with a high level of statistical confidence and targeted performance. All of the experimental conditions were optimized using D-optimal design as a RSM to select the optimal parameters. In addition, this work includes a graphical representation of the relationships between various variables that can greatly affect the results and the intensity of the synchronous fluorescence.

## Abstract 2

In this work, the quantitative determination of an erectile dysfunctional drug avanafil in the presence of its acid-induced degradation product was achieved *via* the application of a pre-optimized novel spectrofluorimetric method.

## Graphical abstract

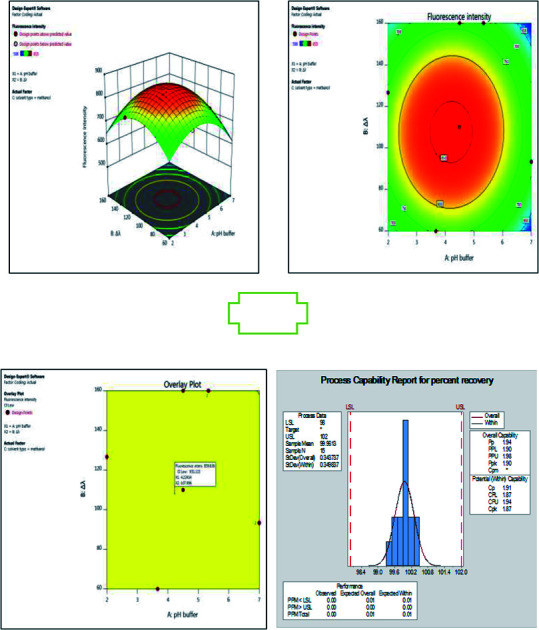

## Introduction

**Fig. 1:**
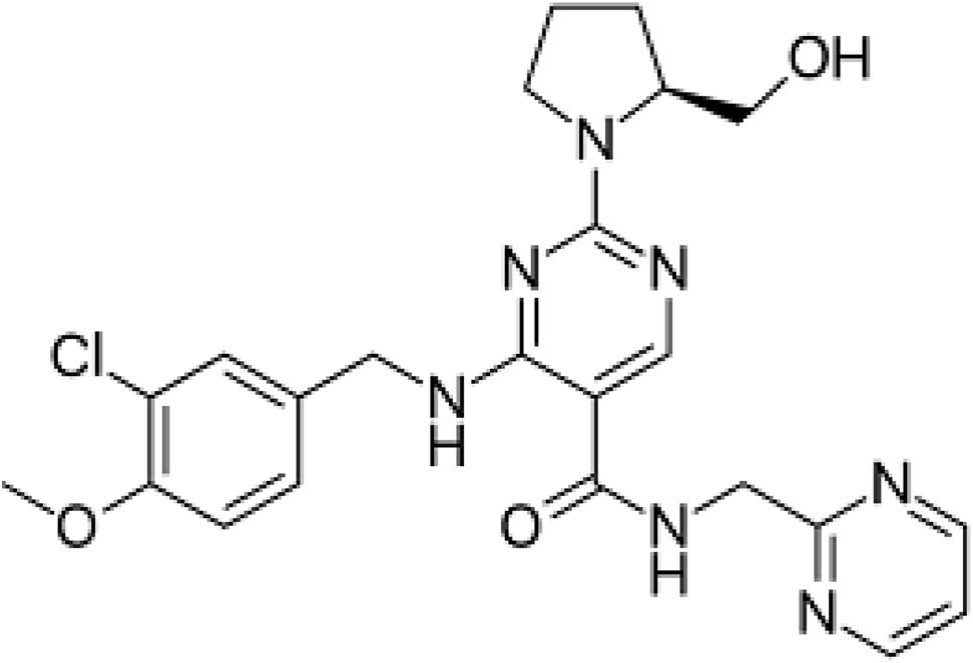
Structural formula of avanafil.

Avanafil is known chemically as (*S*)-4-[(3-chloro-4-methoxybenzyl)amino]-2-[2-(hydroxymethyl)-1-pyrrolidinyl]-*N*-(2-pyrimidinylmethyl)-5-pyrimidinecarboxamide (Fig. 1). It is a white crystalline powder, with the molecular formula C_23_H_26_ClN_7_O_3_ and a molecular weight of 483.95. Additionally, it is soluble in 0.1 M hydrochloric acid, practically insoluble in water and slightly soluble in ethanol. Avanafil is used for the treatment of erectile dysfunction owing to its inhibiting effect on specific phosphodiesterase type 5 (PDE_5_) enzymes present in various body tissues, which are particularly found in the corpus cavernosum in the penis. The advantage of avanafil is that it has a very fast onset of action compared with other PDE_5_ inhibitors (sildenafil, tadalafil and vardenafil). Within fifteen minutes, the patient is able to perform in sexual activity owing to its higher absorption following oral administration. In terms of overdose, avanafil may cause vision changes, sudden vision or hearing loss, shortness of breath and palpitations. Inappropriate storage may cause degradation of the drug, which may contribute to and exaggerate these side effects. However, in spite of these side effects, its use is still preference over the other PDE_5_ drugs.^1–5^ For the determination of avanafil, the literature reveals that many methods have been reported for the quantitative estimation of the studied drug avanafil including multivariate chemometric methods^6^ and different chromatography techniques.^7–12^

Fluorescence spectroscopy is associated with major advantages such as good selectivity, low detection limit and hence high sensitivity. Many drugs have been determined using this technique.^13–19^

Synchronous fluorescence spectroscopy (SFS) was firstly described by Lloyd,^20^ and has numerous advantages over traditional fluorescence, with advantages including a contracted spectral range, narrow spectral bands and simplified emission spectra.^21^

Since there are a lot of variable factors and values of each variable, the efforts and time consumed to optimize them will be considerable until optimal values are reached that lead to ideal results for the method. To this aim, we turned to fractional factorial designs [FrFD], a powerful tool that has the ability to theoretically check all variables and values without them being practically determined. By applying this tool significant values of variables can be selected.

The equation that represents the total number of runs is *N* = 2^*k*−*p*^*,* where *N* is the number of levels of each factor investigated, *k* is the number of factors investigated, and *p* describes the size of the fraction of the full factorial used. Using the adjusted settings of the experiment, a FrFD was used to check the significant relationships between many factors and the responses.^22,23^

After the successful application of FrFD we started to utilize response surface methodology (RSM) to optimize all of the experimental parameters.^24,25^ D-optimal designs are successfully used for decreasing the number of design runs and qualitative factors that have a number of levels above two. The D-optimal design in our experiment has the prime advantage of decreasing the number of runs into just 17 experiments.^26^

In the past few years, Lean Six Sigma (LSS) has affirmed its effectiveness in developing its shareholders and bottom lines through a quality enhancement approach owing to its recognition by greater numbers of corporations. LSS depends on a collaborative team effort to enhance the performance of a method. LSS not only presents a framework for an overall organizational change in culture, but also decreases process defects and waste.^27,28^ The process capability (*C*p) and process capability index (*C*_pk_) have been explored as short-term potential capability statistical tools measures for process capability. Different capability processes use LSS as a common mathematical framework to describe process quality in terms of sigma. In other words, in terms of different processes, it allows us to compare apples to oranges. As we have just seen, the performance concept is incorporated to achieve competitive capacity, efficacy, efficiency, productivity and performance.^29,30^

Many mixtures in their dosage forms and biological fluids samples have been measured and quantified *via* the aid of derivative SFS.^31–36^

The aim of the current work is to develop an accurate, specific, sensitive and capable 2nd derivative SFS method for the determination and resolution of avanafil in pure and dosage forms using a highly optimized area *via* an experimental design approach augmented with LSS as a collaborative process to improve method capability and performance.

## Experimental

### Instrumentation

The instruments and software used in this work include a Jasco spectrofluorometer FP-6200 (Japan); a hot plate (Torrey pines Scientific, USA); a UV lamp (Germany); a Jenway pH meter (USA); a Scilogex-RE 100-pro (USA) rotary evaporator; Minitab® 18.1.0 software, which was used to process capability and performance; and Design-Expert® trial version 11.0 software, which was used for designing and optimizing the proposed method.

## Materials and reagents

### Pure samples

Avanafil (99.48%), prepared using a reported method,^10^ was supplied by Atco Pharma for Pharmaceutical Industries, Egypt.

### Pharmaceutical formulation

Atconafil® 200 mg tablets (batch no. 180667) were used in this work, supplied by Atco Pharma, Egypt.

### Chemicals

Analytical grade chemicals were used in this work, including sodium dodecyl sulphate (SDS) and cetyltrimethylammonium bromide (CTAB) (0.5% aqueous solutions for both, Winlab, UK); methyl cellulose, boric acid, glacial acetic acid, ethyl acetate, hydrochloric acid and sodium hydroxide (El Nasr company, Egypt); triethylamine, phosphoric acid and *n*-hexane (Riedel-deHäen, Germany); and Britton–Robinson (BR) buffer solutions (pH from 2 to 9).

### Standard solutions

#### Avanafil standard solution

Avanafil stock standard solution (100 μg mL^−1^) was prepared using methanol as a solvent in which to first dissolve the powder, then this solution was diluted to obtain a 10 μg mL^−1^ working solution of avanafil.

#### Standard solution of the avanafil acid-induced degradation product

Pure avanafil powder (100 mg) was dissolved in 50 mL of methanol, to which 50 mL of 1 N HCl was added, and then this mixed solution was transferred into a 100 mL volumetric flask. Under reflux, the solution was heated for 6 h, after which the solvent was evaporated under vacuum to leave a residue. Methanol (2 × 10 mL) was used for the extraction of the residue obtained. A stock solution of the acid-induced degradation product was obtained *via* filtration and the addition of 100 mL of methanol. A working solution of the avanafil acid-induced degradation product (10 μg mL^−1^) was prepared *via* further dilution with methanol.

### Procedures

#### Construction of a calibration curve

Aliquots of the working solution of avanafil (10 μg mL^−1^) equal to 0.5–18 μg were transferred into a series of 10 mL volumetric flasks, then 1.5 mL of Britton–Robinson buffer (pH 3) was added before making the volume up to the meniscus using methanol. Synchronous fluorescence spectra were then recorded at Δ*λ* 110 nm. The 2nd derivative spectra of the synchronous fluorescence spectra were obtained by derivation. The 2nd derivative peaks amplitudes were measured at 377 nm. In parallel, a blank experiment was carried out. The calibration graph was obtained by plotting the concentrations of the drug *vs.* the 2nd derivative spectra amplitudes.

#### Synthetic mixture procedure

The previously mentioned procedure was repeated utilizing aliquots of both the avanafil working solution and aliquots of the solution of the avanafil degradation product. Avanafil concentrations were calculated using the obtained regression equation.

### Optimization of the experimental conditions

#### Sequential relationship approach

The response of the critical method parameters (CMPs), including the type of surfactant, pH of the buffer solution, type of solvent, Δ*λ* to be selected and scan width were investigated using FrFD, which was initially run to measure the impacts of the five parameters on the critical method attributes (CMA) (Table 1S†). The number of runs in the experimental matrix was 16 (Table 2S†). CMPs were selected using analysis of variance (ANOVA, Table 3S†), then, the experimental domain was generated (Table 4S†) and the 17 experiments were executed and their responses collected (Table 5S†). Three factors were subjected to analysis, including constant Δ*λ*, buffer solution pH and type of solvent.

The fitted model was obtained using Design-Expert® trial version 11.0 software utilizing a full array of responses (Table 6S†). Statistics of the chosen model were determined using specific software, then the gathered data were subjected to further processing for further optimization. To investigate each factor significance, ANOVA was used (Table 7S†).

#### Analysis of the finished product

Ten Atconafil® tablets (200 mg per tablet) were weighed and finely powdered. An amount equal to 10 mg of avanafil was accurately weighed and then added to a 100 mL volumetric flask and then methanol was used to make the flask volume up to the meniscus.

The solution was subjected to 15 minutes of shaking, 30 minutes of sonication and was then finally filtered. Methanol was used to make the volume up to the meniscus to obtain a solution equivalent to 100 μg mL^−1^, which was then further diluted to obtain a 10 μg mL^−1^ solution. By repeating this general procedure, the tablet content of avanafil was determined from the corresponding regression equation.

## Results and discussion

The main aim of this study was to develop a specific and sensitive 2nd derivative SFS method to determine avanafil in pure and dosage forms without them being previously separated.

### Degradation of avanafil

**Scheme 1:**
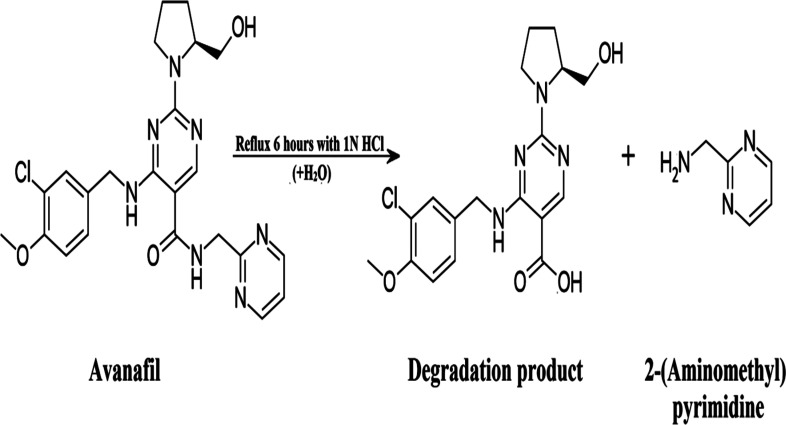
The proposed degradation pathway of avanafil.

Heating avanafil under aqueous, oxidative or basic conditions does not lead to the complete degradation of the drug, whereas refluxing it for 6 h in 1 N HCl leads to its complete degradation, as shown in Scheme 1. Thin layer chromatography (TLC) was used to test the obtained solution. Separation was done using ethyl acetate : glacial acetic acid : methanol (7 : 0.5 : 2.5, by volume) as a mobile phase and ultraviolet (UV) detection at 286 nm, which confirmed the different retention factor (*R*_f_) values at which the spots appeared, with an *R*_f_ of 0.25 for avanafil and 0.63 for its acid-induced degradation product.

### Confirmation of the acid-induced degradation product using infrared (IR) spectroscopy

**Fig. 2:**
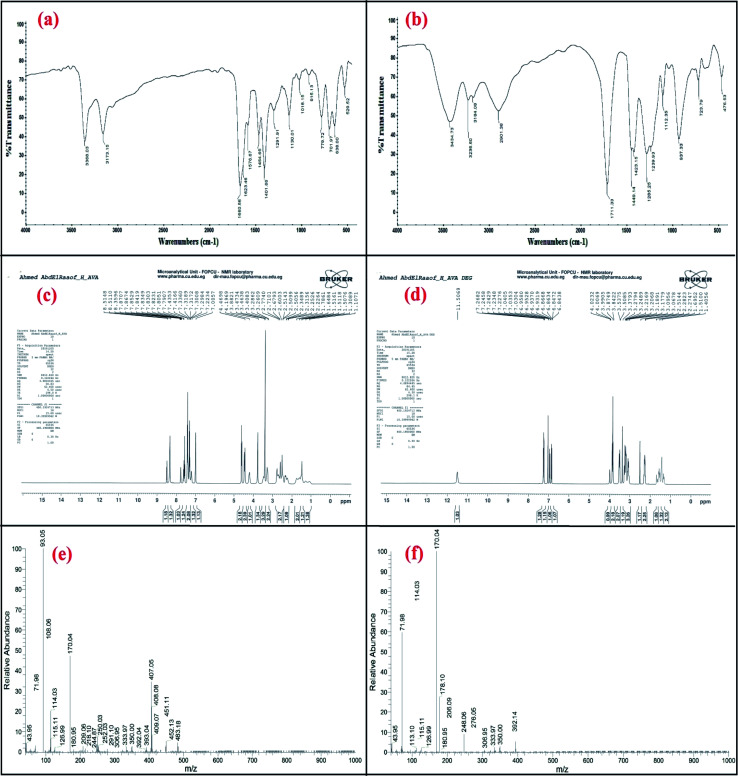
Confirmation of the acid-induced degradation product using (a and b) IR spectroscopy, (c and d) ^1^H NMR spectroscopy and (e and f) mass spectrometry.

The IR spectrum of avanafil in Fig. 2a shows a peak for the carbonyl group of the amide bond (O=C–NH) at 1660.56 cm^−1^, while the IR spectrum of the acid-induced degradation product in Fig. 2b shows the shifting of the (C=O) stretch to 1711.33 cm^−1^, indicating the cleavage of the amide linkage and the formation of a carboxyl group (O=C–OH).

### Confirmation of the acid-induced degradation product using ^1^H nuclear magnetic resonance (NMR) spectroscopy

The ^1^H NMR spectra of the intact avanafil and its acid-induced degradation product in dimethyl sulfoxide (DMSO) reveal the disappearance of the signal of the amide group proton (O=C–NH) at 7.8707 ppm and the appearance of the signal of the carboxyl group proton (O=C–OH) at 11.5069 ppm in the avanafil acid-induced degradation product, as shown in Fig. 2c and d.

### Confirmation of the acid-induced degradation product using mass spectrometry

In the mass spectra, molecular ion peaks at *m*/*z* = 483.18 and *m*/*z* = 392.14 can be observed for avanafil and its acid-induced degradation product, respectively, indicating that the molecular weight of the acid-induced degradation product is 392.14, as shown in Fig. 2e and f.

### Spectral characteristics

**Fig. 3:**
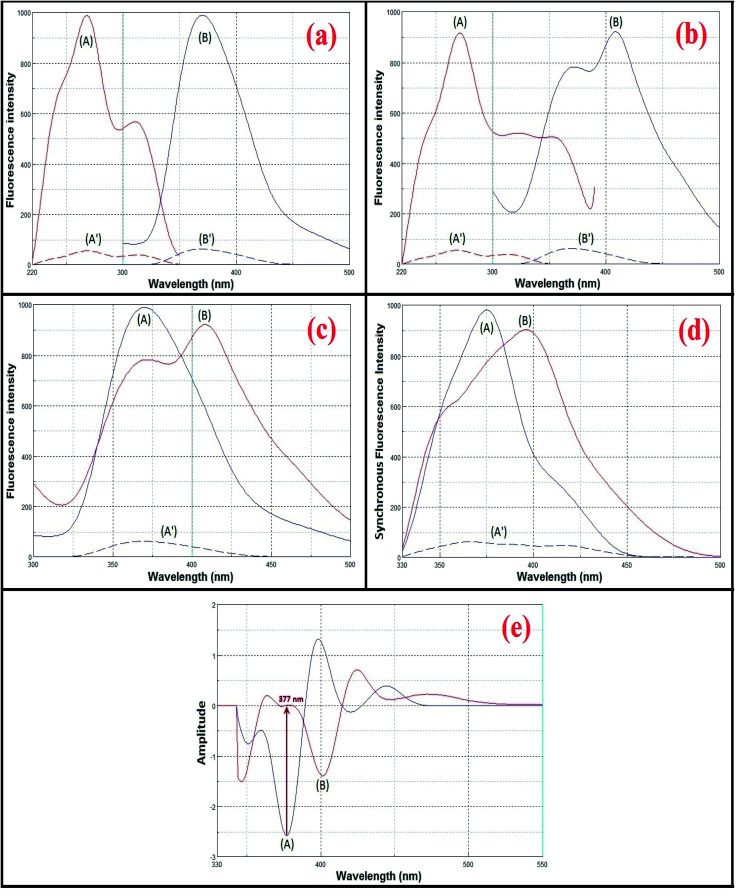
Spectral characteristics of avanafil (A) and its acid-induced degradation product (B).

Both avanafil and its acid-induced degradation product show inherent fluorescence. The fluorescence emission spectra for avanafil and its acid-induced degradation product were observed at 370 and 407 nm, after excitation at 268 and 271 nm, respectively, as shown in Fig. 3a and b. There is heavy overlapping of the emission spectra of avanafil and its acid-induced degradation product (Fig. 3c), which prevents the accurate analysis of avanafil in the presence of its acid-induced degradation product *via* direct determination developed on the basis of their inherent fluorescence. Such a problem cannot be solved using synchronous method entirely, as shown in Fig. 3d. Deriving the 2nd derivative of the synchronous spectra SFS (Δ*λ* = 110 nm) may resolve this difficulty. The 2nd derivative SFS shows a zero-crossing point with fair separation at 377 nm, which allows easy and accurate evaluation of avanafil in the presence of its acid-induced degradation product (Fig. 3e).

### Optimization of the experimental conditions

#### Optimization of the sequential strategy

To determine the optimal conditions using a sequential strategy, first of all, the number of tests must be decreased by targeting the factors that are significant *via* a screening design using FrFD, then RSM can be used to obtain the ideal parameters.^37^

#### Screening design

The screening design gave an *F* value of 30.74, which indicates the significance of the model (Table 3S†). Also, FrFD was used to search for the factors that mostly affect the synchronous fluorescence intensity (SFI) and it was found that the type of solvent, constant wavelength Δ*λ* and pH of the buffer solution were non-zero, which indicates statistical significance (Fig. 1S†). The type of the surfactant and scanning width have no significant effect, so the type of solvent, constant Δ*λ* and pH of the buffer were then processed using RSM.

#### D-optimal design approach

The quadratic model looks best upon running the Design-Expert® trial version 11.0 software (and is highlighted by a bold line of text in Table 6S†). The analysis of the data gathered from the D-optimal design show how important it is to add quadratic terms to this model (Table 7S†). To construct the predictive models and correlate the selected parameters to the values of their responses, the data were represented by a quadratic equation.

For the SFI, the mathematical prediction model was calculated as follows(SFI) = 842.73 − 15.62*x*_1_ − 9.32*x*_2_ − 15.18*x*_3_ − 110.44*x*_1_^2^ – 110.62*x*_2_^2^ + 10.12*x*_1_*x*_3_where *x*_1_ is the buffer solution pH, *x*_2_ is the constant wavelength Δ*λ*, and *x*_3_ is the type of solvent, respectively.

The *F* value of the probability falls below 0.05, which indicates that there is no significance in the lack of fit, as shown in the ANOVA table of the quadratic model (Table 8S†). The difference between the predicted *R*^2^ and the adjusted *R*^2^ is less than 0.2, where the predicted *R*^2^ of 0.9613 is in reasonable agreement with the adjusted *R*^2^ of 0.9884. Additionally, the Adeq precision is 42.61 in adequate value as adequate precision means that the signal-to-noise ratio should be greater than 4 to be agreeable (Table 8S†). The prediction residual sum of squares exhibits a high *R*^2^ and low standard deviation values for the quadratic model.

#### Diagnosis of the statistical properties of the fitted model

The data points are almost linear in the residuals probability curve of the determination of avanafil. Residuals calculated externally can expose the outliers, revealing that no outliers are present and that the residuals are randomly distributed according to the run number. From the plot of the residuals *vs.* the predicted values, it is clear that the residuals are not dependent on the predicted values. Also, the Box Cox plot shows that there is no need for further transformation, ensuring that the regression model accuracy outcomes are acceptable (Fig. 2S†).

#### Setting the optimization criteria

Design-Expert® trial version 11.0 software was used to set the maximum values of the SFIs. For any given response, the values of desirability fall between 0 and 1. The software searches for the highest total desirability by combining each desirability into one value.^38^ The most desirable one will be highlighted by the software.

Contour desirability, overlay plots, 3D plots and numerical optimization show that a desirability of close to 1 can be achieved utilizing a buffer with a pH of 4.2, methanol as a solvent and a constant Δ*λ* of 107 nm, but the selected wavelength was 110 nm because it offers the best synchronous spectrum with fair separation at Δ*λ* = 110 nm leading to an improvement in the sensitivity and resolution of the method (Fig. 3S–6S†), which is shown in the data in Table 9S.†

#### Process capability and performance



1

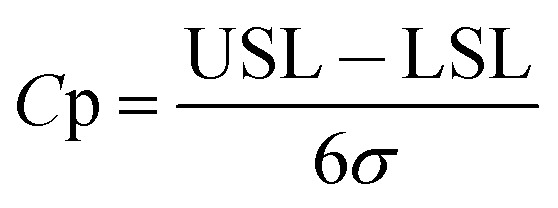


*C*p is a measure of the ability of a process to induce the output within specification limits regardless of whether it is in the center or not by estimating the ratio of the process specification range to six-sigma . The process capability index (*C*_pk_) is a measure of how centred the data is between the specification limits. Data was collected from the percentage recovery of different avanafil concentrations measured using the Minitab® 18.1.0 software.

**Fig. 4:**
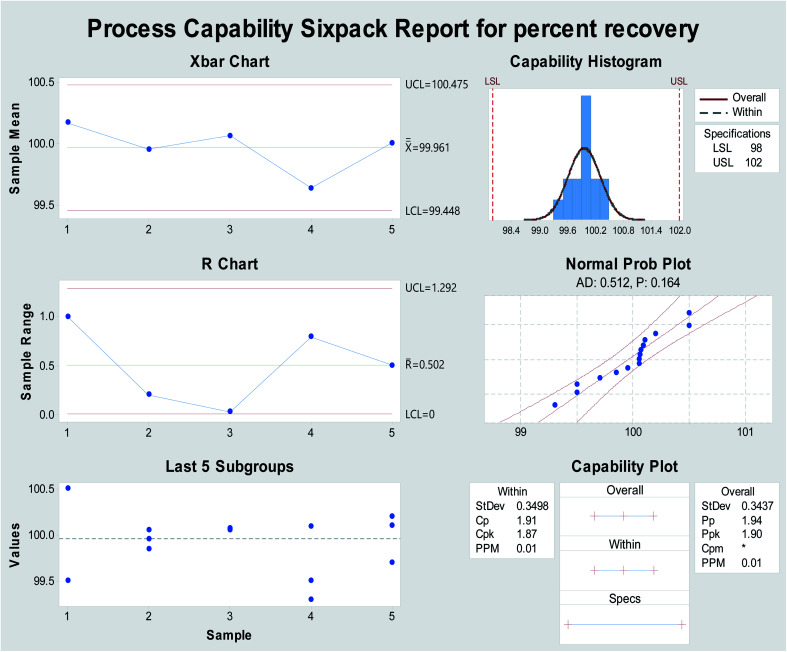
Process capability six-pack quality tools for obtaining the normally distributed assay results of percentage recovery using Minitab® 18.1.0.

As the process capability improves, the variability decreases, and the data will be more tightly centered between the specification limits (Fig. 4). The current work was used to analyze and assure that the process capability index (*C*_pk_) is > 1.33. Process capability six-pack analysis and the process capability report indicated that the reset of all of the measurements within the acceptable specification range is a 6-sigma capable process and that the data are statistically capable and more accurate (Fig. 4 and 7S†), as presented in Table 1.

**Table 1:** Application of quality control and statistical tools for process capability and performance^a^

Parameters	Proposed method
LSL	98
USL	102
Mean	99.96
SD	0.349
Sample number	15
*C*_p_	1.91
*C*_PU_	1.94
*C*_PL_	1.87
*C*_pk_	1.94
*P*_P_	1.90
*P*_pk_	

a LSL: lower specification limit, USL: upper specification limit, *C*_p_: process capability, *C*_PU_: process capability within USL, *C*_PL_: process capability within LSL, *C*_pk_: process capability centered, *P*_P_: process performance and *P*_pk_: process performance centered.

## Method validation^39^

### Linearity

The amplitude of the 2nd derivative SFS at 377 nm *versus* the concentrations was plotted to obtain the method calibration graph, with the data listed in Table 2.

**Table 2:** Spectral data of the estimation of avanafil using the proposed method

Parameters	Proposed method
Linearity range (μg mL^−1^)	0.05–1.8
Slope	1.1752
Intercept	0.4776
LOD (μg mL^−1^)	0.01
LOQ (μg mL^−1^)	0.04
Correlation coefficient (*r*)	0.9994
Accuracy (% *R*)^a^	99.13
Precision (% RSD)^b^	
Repeatability	0.729
Intermediate precision	1.054

a Nine average determinations.

b Nine precision determinations.

### Limits of detection (LOD) and quantitation (LOQ)

LOD and LOQ values were calculated and the results were listed in Table 2.

### Accuracy and precision

Agreeable values of % recovery (%*R*) assure the accuracy of the method, as listed in Table 2. Also, elevated method precision was assured by low values % RSD listed in Table 2.

### Specificity

The proposed method shows high specificity for the determination of avanafil in the presence of its acid-induced degradation product, as shown in Table 3. Also, we used a standard addition technique as a specificity measure and the values listed in Table 4 indicate no interference from the matrix.

**Table 3:** Application of the proposed method for the analysis of avanafil in mixtures alongside its acid-induced degradation product

Intact (μg ml^−1^)	Acid-induced degradation product (μg ml^−1^)	Intact found (μg ml^−1^)	% Recovery of intact
1.5	0.3	1.523	101.53
1.3	0.5	1.310	100.77
1.1	0.7	1.088	98.91
0.9	0.9	0.913	101.44
0.7	1.1	0.699	99.86
0.5	1.3	0.503	100.60
0.3	1.5	0.298	99.33
0.2	1.6	0.197	98.50
Mean ± % RSD	100.12 ± 1.144		
SD	1.147		

**Table 4:** Application of the standard addition technique for study of the matrix effect

Atconafil® tablets taken (μg mL^−1^)	Pure added (μg mL^−1^)	Pure found (μg mL^−1^)	% Recovery
0.4	0.2	0.196	98.00
	0.4	0.407	101.75
	0.6	0.604	100.67
Mean ± % RSD	100.20 ± 1.788		
SD	1.793		

### Application to the finished product

The avanafil in 200 mg Atconafil® tablets was determined using the proposed method. The obtained results show the absence of any interference from either excipients or additives. The results of the reported method^10^ were compared with those of the proposed method. The proposed method shows good accuracy and precision for the assay of avanafil in Atconafil® tablets, with the relevant values listed in Table 5.

**Table 5:** Results obtained after the determination of avanafil in 200 mg Atconafil® and comparison with the reported method

Parameter	Proposed method	Reported method^10^
*n* a	5	5
% *R*	99.87	99.44
% RSD	1.145	0.940
SD	1.142	0.935
Variance	1.308	0.874
Student's *t*-test (2.306)^b^	0.651	—
*F* value (6.388)^b^	1.496	—

a Number of experiments.

b Tabulated values of “*t* “and “*F*” at (*P* = 0.05).

## Conclusion

The proposed method utilizes experimental design augmented with LSS as a powerful tool for the estimation of avanafil in the presence of its acid-induced degradation product with high accuracy and precision, with fewer experiments and at low cost without any interference. The augmentation of using theoretical software alongside practical study is a promising approach by which to achieve analysis with high capability and performance. The whole procedure was checked for validity and found to be valid in various quality control laboratories.

## Conflicts of interest

The authors declare that they have no conflict of interest.

## Supplementary Material

RA-011-D0RA08216C-s001
