# Supplementary material for: Second derivative synchronous fluorescence determination of avanafil in the presence of its acid-induced degradation product aided by powerful Lean Six Sigma tools augmented with D-optimal design
Source: RSC Adv. 2021 Jan 19;11(7):3834–42. doi: 10.1039/d0ra08216c (PMC8694234; doi:10.1039/d0ra08216c)
Supplement: RA-011-D0RA08216C-s001 [file RA-011-D0RA08216C-s001.pdf]

Electronic Supplementary Material (ESI) for RSC Advances.
This journal is © The Royal Society of Chemistry 2020

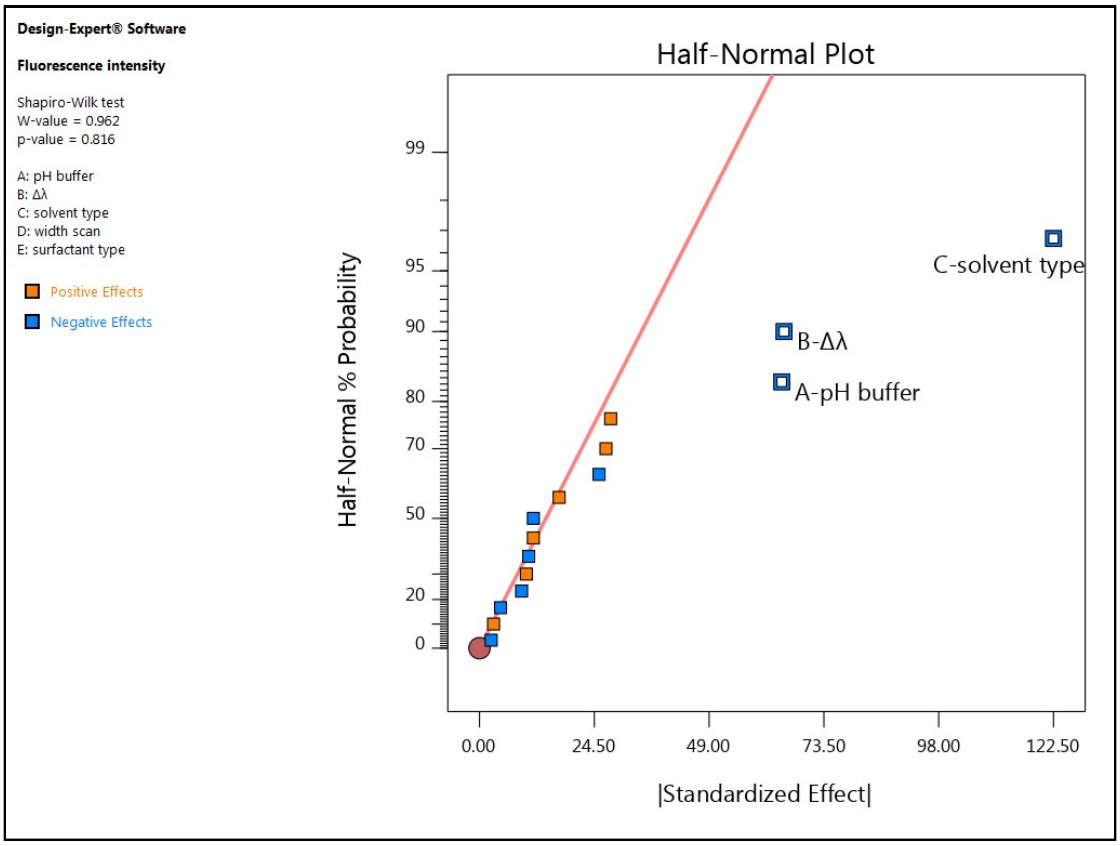

Fig. 1S: Half normal plot of all factors estimated effect.

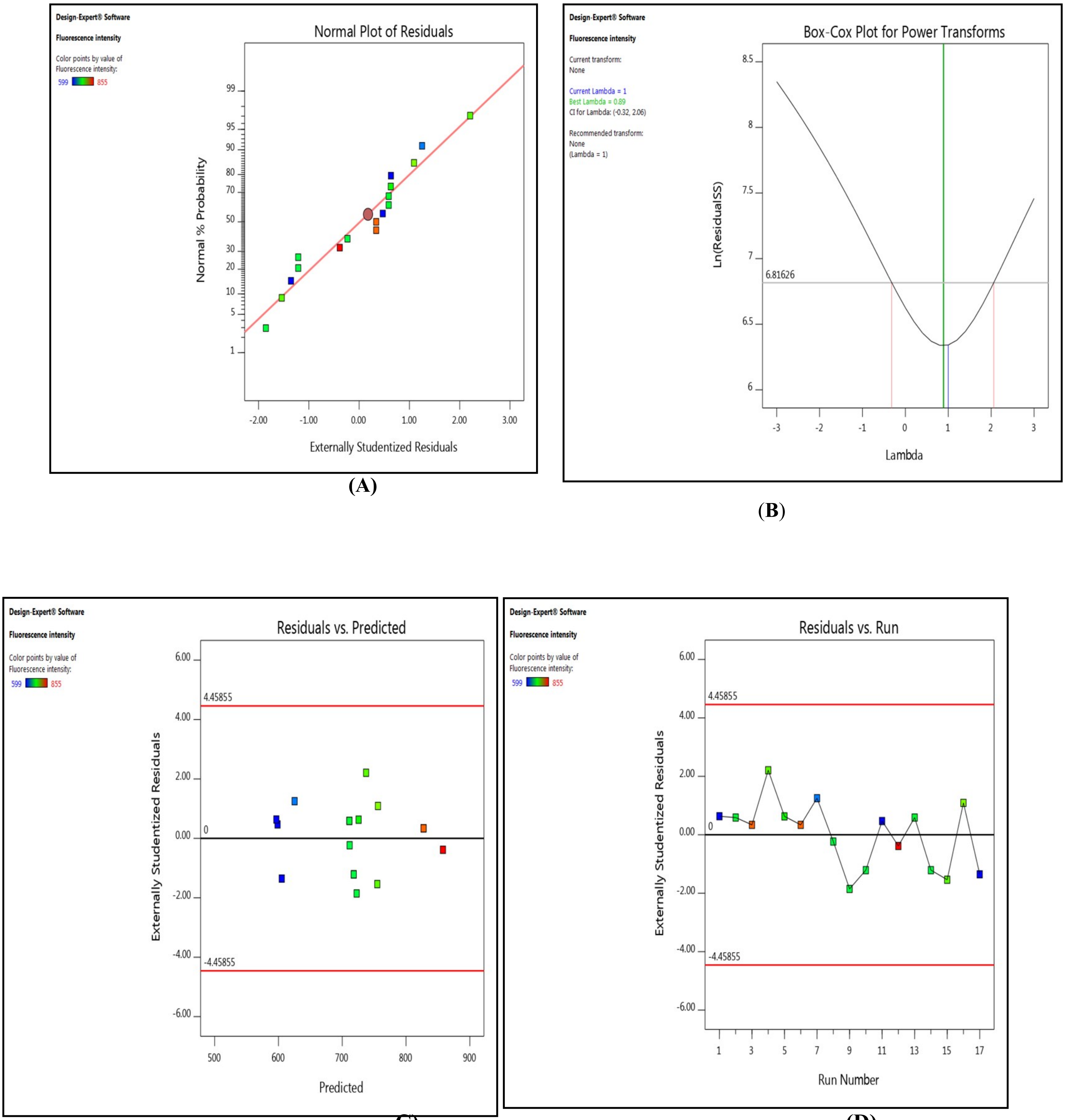

(A)

(B)

C)

(D)

(

Fig. 2S: Normal probability plot of the residuals (A), Box-cox plot (B), residuals versus predicted (C), residuals versus run (D).

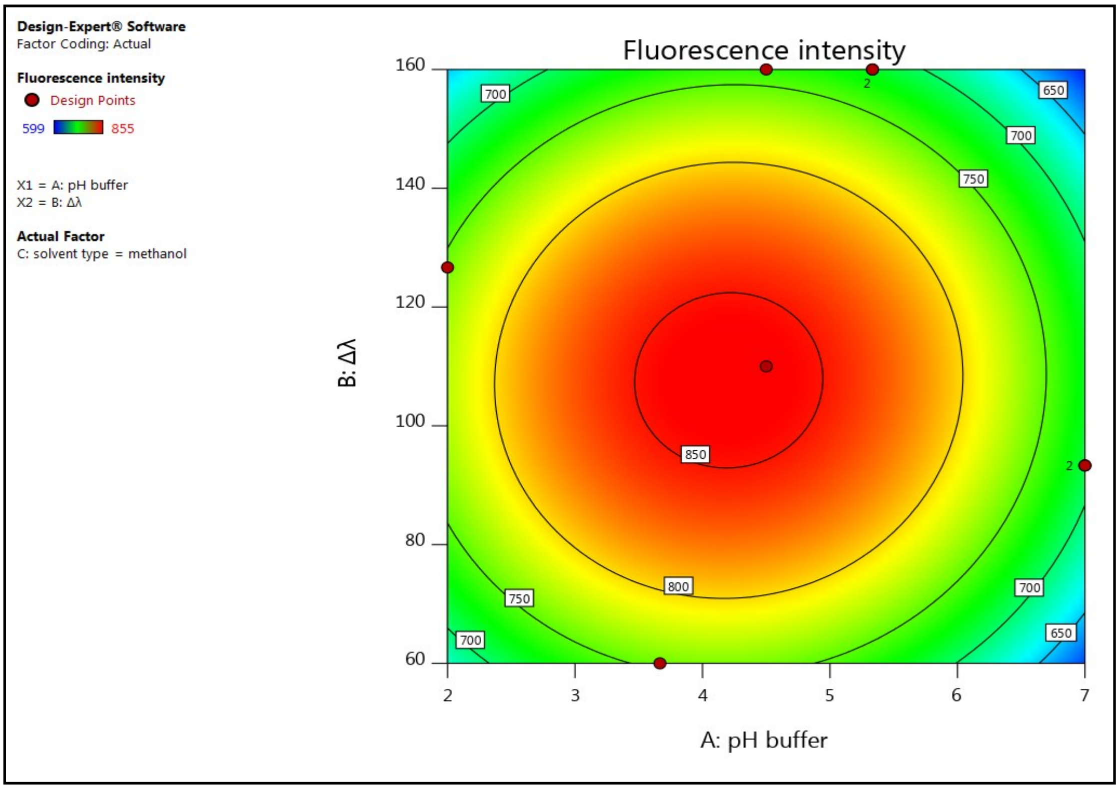

Fig. 3S: Contour desirability plot.

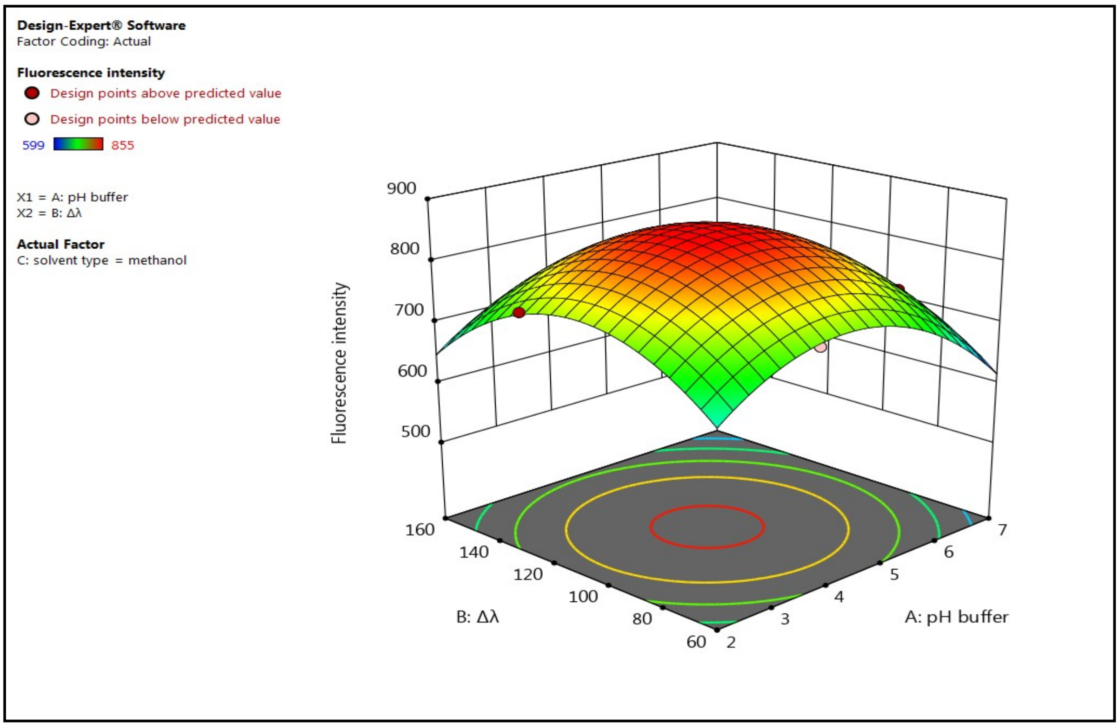

Fig. 4S: 3D desirability plot.

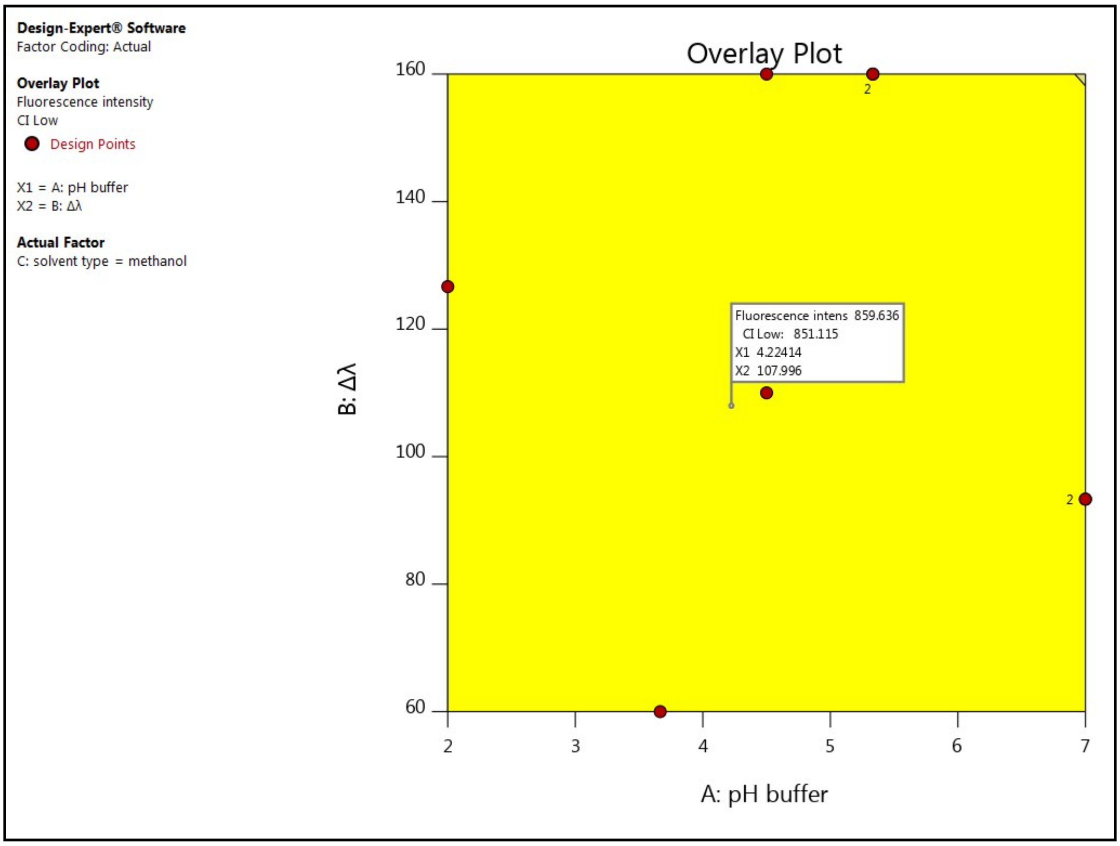

Fig. 5S: overlay desirability plot.

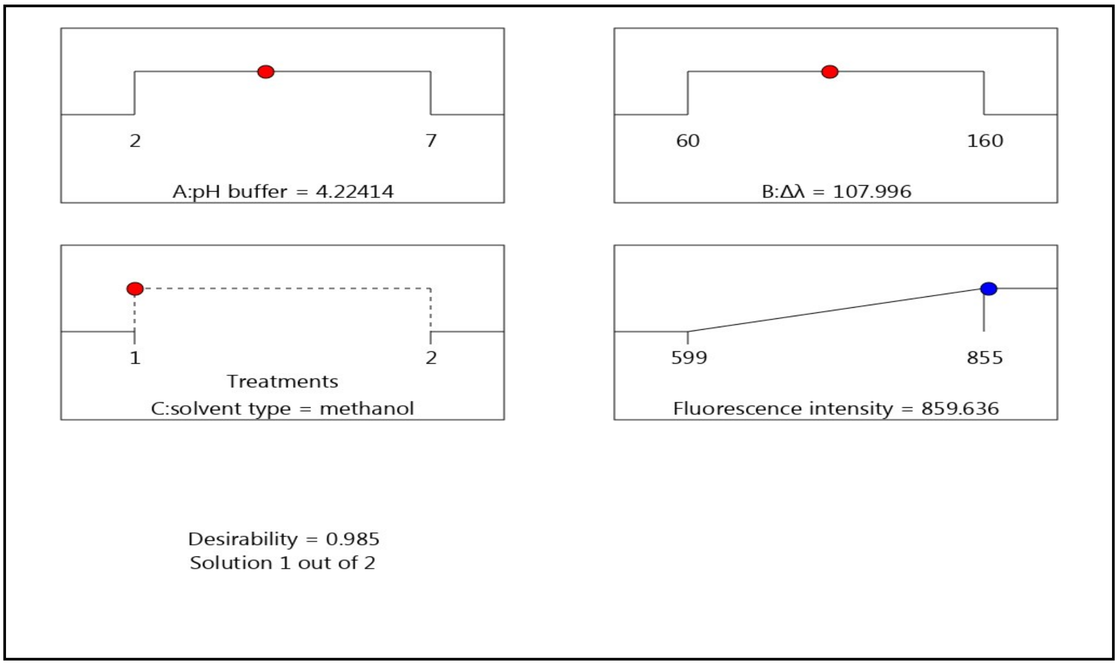

Fig. 6S: Numerical optimization ramps view for solutions.

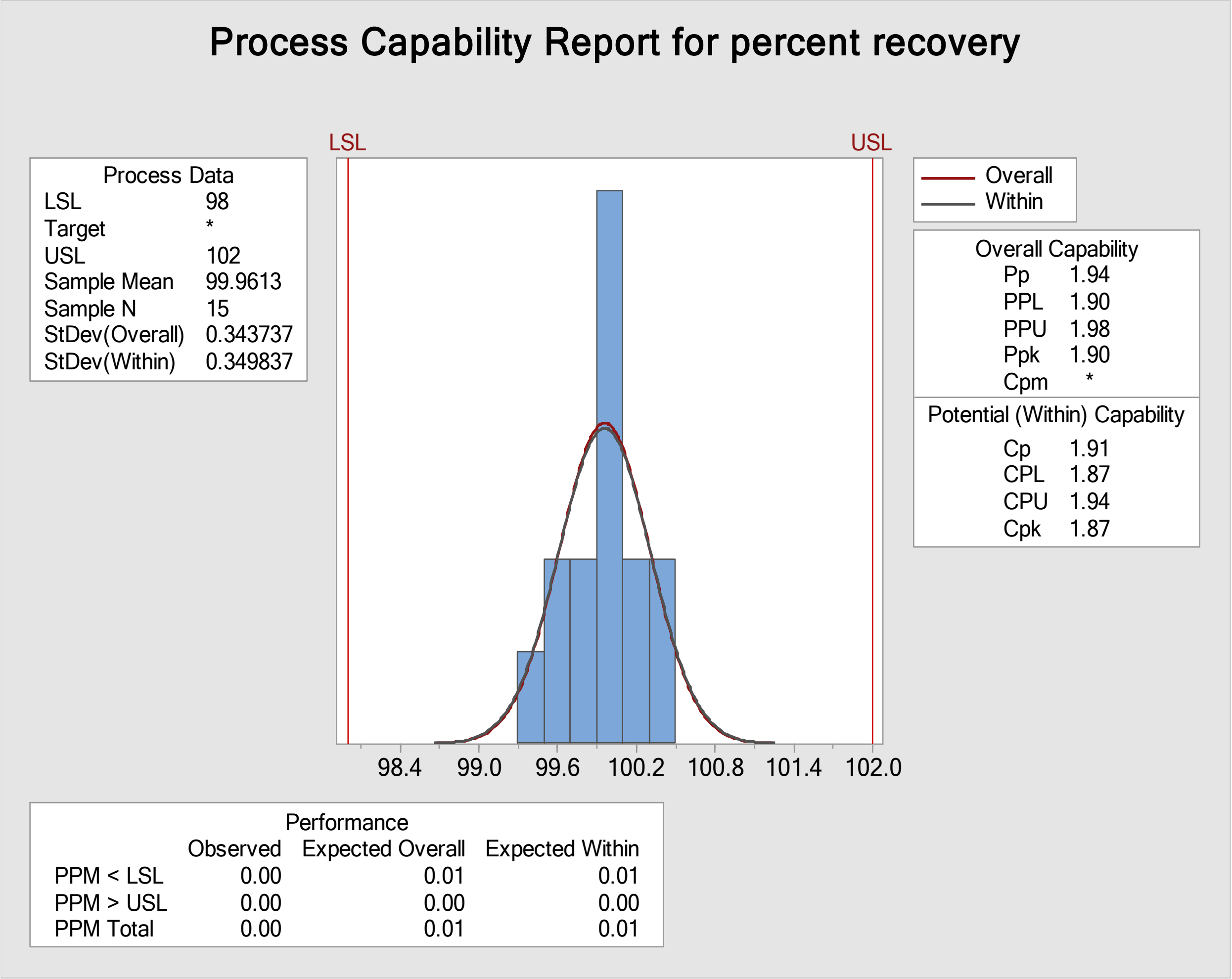

Fig.7S: Process capability and performance for the recovery percent of the proposed method.

Table 1S: Build information of variables specification and experimental domain factors in fractional factorial design

Factor	Name	Type	Minimum	Maximum	Coded Low (-1)	Coded high (+1)
A	pH buffer	Numeric	2.00	6.00	2.00	6.00
B	Δλ	Numeric	60.00	160.00	60.00	160.00
C	solvent type	Categoric	Methanol	Ethanol	Methanol	Ethanol
D	width scan	Numeric	5.00	10.00	5.00	10.00
E	surfactant type	Categoric	SDS	CTAB	SDS	CTAB

Table 2S: fractional factorial design for experimental variables and synchronous fluorescence intensity response

Run	Factor 1	Factor 2	Factor 3	Factor 4	Factor 5	Response 1
	A:pH buffer	B:Δλ	C:solvent type	D:width scan	E:surfactant type	synchronous Fluorescence intensity
1	2	60	Ethanol	5	SDS	710
2	2	160	Ethanol	10	SDS	650
3	2	160	Ethanol	5	CTAB	678
4	6	160	Ethanol	10	CTAB	620
5	6	160	Methanol	5	CTAB	730
6	2	60	Methanol	10	SDS	870
7	6	60	Methanol	10	CTAB	760
8	6	160	Ethanol	5	SDS	618
9	2	160	Methanol	5	SDS	760
10	2	60	Ethanol	10	CTAB	710
11	2	160	Methanol	10	CTAB	850
12	2	60	Methanol	5	CTAB	866
13	6	60	Ethanol	10	SDS	670
14	6	60	Methanol	5	SDS	820
15	6	160	Methanol	10	SDS	670
16	6	60	Ethanol	5	CTAB	690

Table 3S ANOVA for selected fractional factorial model

Source	Sum of Squares	df	Mean Square	F-value	p-value	
**Model**	93566.00	3	31188.67	30.74	< 0.0001	significant
A-pH buffer	16641.00	1	16641.00	16.40	0.0016	
B-Δλ	16900.00	1	16900.00	16.66	0.0015	
C-solvent type	60025.00	1	60025.00	59.17	< 0.0001	
**Residual**	12174.00	12	1014.50			
**Cor Total**	1.057E+05	15				

The **Model F-value** of 30.74 implies the model is significant. There is only a 0.01% chance that an F-value this large could occur due to noise.

P-values less than 0.0500 indicate model terms are significant. In this case A, B, C are significant model terms.

Table 4S: Build information of variables specification and experimental domain factors in D-optimal design

Factor	Name	Minimum	Maximum	Coded Low (-1)	Coded high (+1)	Mean Centre point (0)
A	pH buffer	2.00	7.00	2.00	7.00	4.50
B	Δλ	60.00	160.00	60.00	160.00	110
C	solvent type	methanol	ethanol	methanol	ethanol	methanol

Table 5S: D-optimal design for experimental variables and synchronous fluorescence intensity response

	Factor 1	Factor 2	Factor 3	Response 1
Run	A:pH buffer	B:Δλ	C:solvent type	Fluorescence intensity
1	7	160	ethanol	599
2	7	93.3333	methanol	715
3	4.5	110	ethanol	830
4	4.5	160	methanol	750
5	4.5	60	ethanol	730
6	4.5	110	ethanol	830
7	2	60	ethanol	630
8	7	110	ethanol	710
9	2	110	ethanol	712
10	5.33333	160	methanol	710
11	2	160	ethanol	600
12	4.5	110	methanol	855
13	7	93.3333	methanol	715
14	5.33333	160	methanol	710
15	3.66667	60	methanol	750
16	2	126.667	methanol	760
17	7	60	ethanol	600

Table 6S: fitting summary for the suggested experimental design.

**Source**	**Sequential p-value**	**Lack of Fit p-value**	**Adjusted R^2**	**Predicted R^2**	
Linear	0.5215		-0.0413	-0.3911	
2FI	0.9744		-0.3256	-3.2080	
**Quadratic**	**< 0.0001**		**0.9884**	**0.9613**	**Suggested**
Cubic			1.0000		Aliased

Table 7S ANOVA for Quadratic model

Source	Sum of Squares	df	Mean Square	F-value	p-value	
**Model**	97244.34	8	12155.54	171.31	< 0.0001	significant
A-pH buffer	1965.79	1	1965.79	27.70	0.0008	
B-Δλ	743.02	1	743.02	10.47	0.0120	
C-solvent type	3295.00	1	3295.00	46.44	0.0001	
AC	825.41	1	825.41	11.63	0.0092	
A^2	38597.70	1	38597.70	543.95	< 0.0001	
B^2	42236.28	1	42236.28	595.23	< 0.0001	
**Residual**	567.66	8	70.96		0.085	insignificant
Lack of Fit	567.66	5	113.53			
Pure Error	0.0000	3	0.0000			
**Cor Total**	97812.00	16				

Factor coding is **Coded**.
Sum of squares is **Type III - Partial**

The **Model F-value** of 171.31 implies the model is significant. There is only a 0.01% chance that an F-value this large could occur due to noise.

P-values less than 0.0500 indicate model terms are significant. In this case A, B, C, AC, A^2, B^2 are significant model terms. Values greater than 0.1000 indicate the model terms are not significant. If there are many insignificant model terms (not counting those required to support hierarchy), model reduction may improve your model.

Table 8S Fit Statistics

**Std. Dev.**	8.42		**R^2**	0.9942
**Mean**	718.00		**Adjusted R^2**	0.9884
**C.V. %**	1.17		**Predicted R^2**	0.9613
			**Adeq Precision**	42.6169

The **Predicted R^2** of 0.9613 is in reasonable agreement with the **Adjusted R^2** of 0.9884; i.e. the difference is less than 0.2.

Adeq Precision measures the signal to noise ratio. A ratio greater than 4 is desirable. Your ratio of 42.617 indicates an adequate signal. This model can be used to navigate the design space.

Table 9S: Solutions determination of optimum response.

Number	pH buffer	Δλ	solvent type	Fluorescence intensity*	Desirability	Desirability (w/o Intervals)	
**1**	**4.226**	**107.974**	**methanol**	**859.635**	**0.985**	**1.000**	**Selected**
2	4.433	107.751	ethanol	827.799	0.863	0.894	
